# Intraoral Scans of Full Dental Arches: An In Vitro Measurement Study of the Accuracy of Different Intraoral Scanners

**DOI:** 10.3390/ijerph20064776

**Published:** 2023-03-08

**Authors:** Giovanni Giuliodori, Giorgio Rappelli, Luca Aquilanti

**Affiliations:** 1 Independent Researcher, Via Brecce Bianche 94, 60131 Ancona, Italy; 2 Department of Clinical Specialistic and Dental Sciences, Università Politecnica delle Marche, Via Tronto 10/A, 60126 Ancona, Italy; 3 Dentistry Clinic, National Institute of Health and Science of Aging, IRCCS INRCA, Via Tronto 10/A, 60126 Ancona, Italy

**Keywords:** intraoral scanner, full dental arch, accuracy, trueness, precision, scanning strategies

## Abstract

The aim of this in vitro study was to evaluate the accuracy of different intraoral scanners (IOS), according to different scanning strategies and to the experience of the operator. Six IOS setups were used in this study. Ten scans of a complete epoxy-resin-made maxillary dental arch were performed with each IOS, using four different scanning techniques (manufacturer-suggested scanning strategy, cut-out rescan technique, simplified scanning technique, novel scanning technique). Scans were also performed by an expert operator in the field of digital dentistry. An operator with no experience in the field of intraoral scans performed 10 scans following each of the scanning strategy suggested by the manufacturer. The master model was scanned with an industrial high-resolution reference scanner to obtain a highly accurate digitized reference model. All the digital models were aligned with the reference model using a software aimed at comparing the STL files. A total of *n* = 300 scans were performed. Once the data were pooled, Medit i700 and Primescan obtained the best results in terms of both trueness and precision, showing no statistically significative differences (*p* > 0.05) to the first and the second scanning technique, Medit i700 scanner allowed to obtain the best values both in terms of trueness (24.4 ± 2.1 μm and 21.4 ± 12.9 μm, respectively) and precision compared to other IOS (23.0 ± 1.6 μm and 30.0 ± 18.0 μm, respectively). When considering the third scanning technique, Medit i700 recorded the best values in terms of trueness while Primescan recorded the best values in terms of precision (24.0 ± 2.7 μm and 26.8 ± 13.7 μm, respectively). When considering the two operators, significant differences between the two were found only with Medit i700 (*p* < 0.001). The examined IOS showed statistically significant differences in terms of trueness and precision. The used scanning strategy is a factor influencing the accuracy of IOS. Considering the expertise of the operators, clinically scanning strategies are not operative sensitive in terms of accuracy.

## 1. Introduction

In recent years, digital technologies have become widely used in dental practice, resulting in a consistent evolution and transformation of clinical workflows. The digitalization process radically changed the dental world, becoming part of it. The use of computer represents the pivotal point of the CAD/CAM technology (Computer-Aided Design/Computer-Aided Manufacturing) that was introduced in dentistry more than fifty years ago. Initially, CAD/CAM technology aimed at carrying out only single restorations. Then, due to technological advances, even complex oral rehabilitations, including more extended fixed dental prostheses, either on natural teeth or implants, were performed [1,2].

This technology was described for the first time in 1973 by Duret, aiming at automating the manufacturing processes of prosthetic products in order to optimize the quality and, thus, increase the efficiency of the entire workflow [3]. In particular, Duret developed and patented a CAD/CAM system that allowed to manufacture a dental crown in 4 h [4]. Since 1980, when the first intraoral scanner (IOS) was made, this technology continued to be developed and, in 1987, the first intraoral scanner (IOS) was introduced in the dental market [5,6]. The digital workflow was implemented earlier in the dental laboratory setting than in the clinical one, through an indirect digitalization process. In particular, the conventional impression performed by the dentist was supposed to be casted in the laboratory to obtain a physical model, then digitalized using an optical scanner. The digitalized model was subsequently processed using CAD/CAM systems [7]. Since then, IOS became very common in dental practice. As a consequence of the massive introduction of IOSs in the dental market, clinicians are now able to start the digital workflow in the dental office. In fact, IOSs have recently benefited significant technological developments from software and hardware point of view. These improvements allowed IOSs to be as accurate as the conventional impressions [8]. In particular, digital scans are a clinically acceptable alternative to conventional impression methods in the fabrication of crowns (both on teeth and implants) and short fixed dental prostheses, while conventional impressions are still recommended for full-arch impressions [9].

The fundamental characteristic of an IOS is, in fact, the accuracy, as a combination of trueness and precision. According to the definition by the International Standard Organization (ISO) 5725:1, trueness is defined as the “closeness of agreement between the arithmetic mean of a large number of test results and the true or accepted reference value” while, precision is defined as “the closeness of agreement between different test results” [10]. Accuracy and precision are essential for obtaining an adequate digital scan and, consequently, an excellent final product.

A substantial role is also played by scanning strategies that may have an impact on the success of intraoral scanning, as they are likely to influence the accuracy of digital scan [11]. Moreover, taking into account the learning curve needed to master intraoral scanners, it could be speculated that the experience of the scanning operator may also play an important role, affecting the accuracy of the digital scan. Additionally, the extension of the area to scan affects the accuracy of the scan itself, as a consequence of the stitching process. In particular, in small segments, such as sextants, the scanning pattern does not affect trueness and precision [12]. When considering full arch scans, significant differences were reported [13,14,15]. However, the improvements introduced by the latest software and scanner versions ensure high levels of accuracy even for full dental arch scans, as reported in the present study [16,17,18,19].

Considering the abundance of IOSs, it becomes crucial to identify which is the most accurate system. This study aims at: evaluating and comparing the accuracy (trueness and precision) of different IOSs; comparing different scanning strategies in terms of accuracy; verifying whether the scanning strategies are operative-sensitive. The null hypotheses were that there would be no significant differences in the digital scan accuracy (trueness and precision) of different intraoral scanners, scanning strategies and operators.

## 2. Materials and Methods

**Figure 1:**
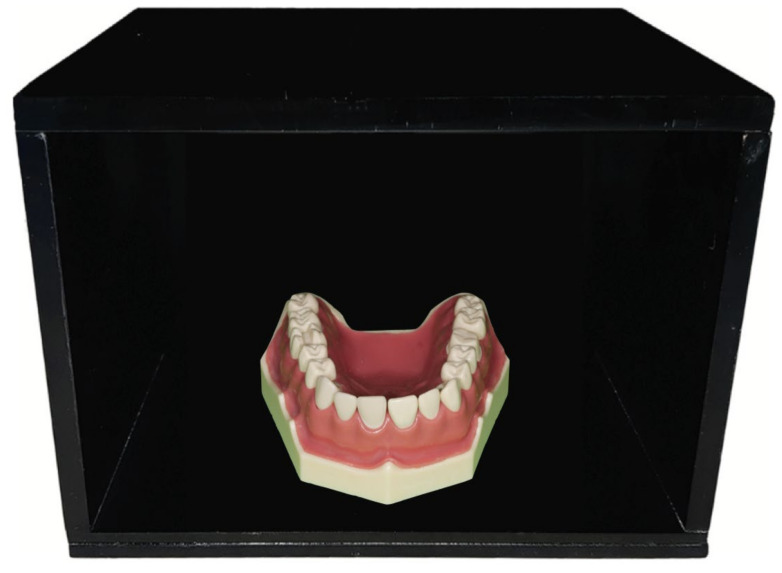
The epoxy-resin-made maxillary dental arch was inserted in an opaque black methacrylate box, in order to simulate oral cavity.

An upper epoxy-resin-made maxillary dental arch was used in this study. Epoxy resin is an opaque and dimensionally stable material with good mechanical and chemical resistance. The scans, using different scanning techniques, were performed for the complete dental arch (CDA), with the master model inserted in an opaque black methacrylate box, in order to simulate oral cavity (Figure 1).

Six intraoral scanner setups were used in this study: AC Omnicam, software v 5.1.3 (Dentsply Sirona, Charlotte, NC, USA); AC Primescan, software v 5.1.3 (Dentsply Sirona, Charlotte, NC, USA); Trios 4, software v 1.7.9.1 (3Shape, Copenhagen, Denmark); iTero Element 5D, software v 2.6.3.369 (Align Technology, San Jose, CA, USA); Dexis IS 3700, software scan flow v 1.3 (Dental Imaging Technologies Corporation, Hatfield, PA, USA); Medit i700, software v. 2.5.7 (Medit, Seoul, Republic of Korea).

**Figure 2:**
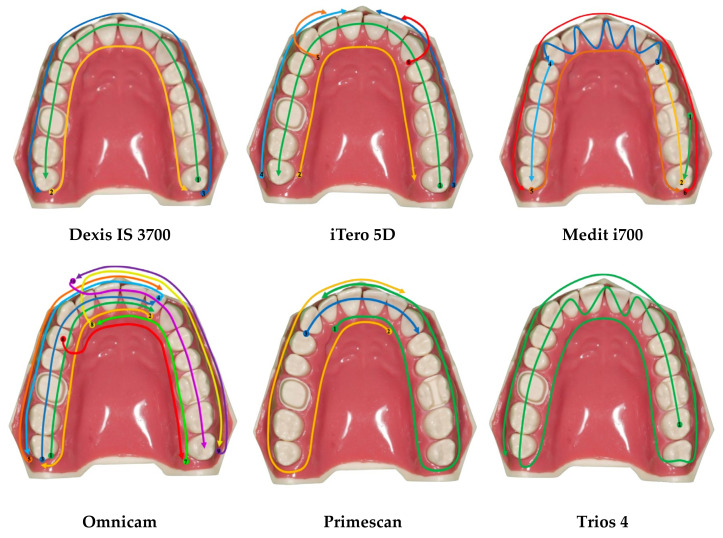
Graphical representation of the scanning strategies recommended by the manufacturers. The colored arrows indicate the sequence of movements of the scanner.

The six intraoral scanners were calibrated according to the manufacturer’s guidelines. A convenience sample size was used. Ten scans of the entire jaw arch were performed with each IOS, using four different scanning techniques for each scanner:

- Technique 1: the strategy recommended by the manufacturer (specific for each different IOSs, Figure 2); in addition, cut-out rescan technique was also performed, cutting out three segments of the dental arch and rescanning them. The procedure began by scanning the cast, and then three delimited areas were sequentially edited using the cut-off tool and then rescanned until the substitution of new images of these areas was complete. In particular, the selection was performed in correspondence of the right maxillary first molar (full crown preparation), the left maxillary first molar (onlay preparation), and the left maxillary central incisor (veneer preparation).
- Technique 2: a simplified scanning technique (briefly, 1. From the palatal surface of the distal right molar to the palatal surface of the distal left molar; 2. From the occlusal surface of the distal left molar to the occlusal surface of the distal right molar; 3. From the buccal surface of the distal right molar to the buccal surface of the distal left molar);
- Technique 3: a new scanning technique proposed by Passos et al. [11] (briefly, 1. From the palatal surface of the distal right molar to the palatal surface of the distal left molar; 2. From the occlusal surface of the distal left molar to the occlusal surface of the distal right molar; 3. From the buccal surface of the distal right molar to the buccal surface of the distal left canine; 4. From the buccal surface of the distal left molar to the buccal surface of the distal right canine).

**Figure 3:**
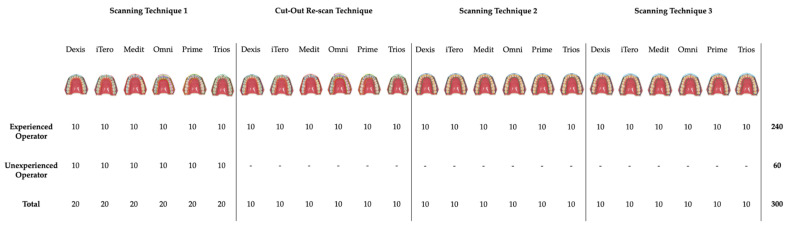
Graphical and synoptic representation of the performed scans per operator. A total of *n* = 300 were obtained and analyzed.

In order to assess if scanning techniques were operative-sensitive, scans were performed by both a trained and expert operator and by an operator with no experience in the field of intraoral scans. The latter performed 10 scans of the master model following each of the scanning strategy suggested by the manufacturer. Figure 3 synoptically shows the performed scans per operator.

The scan data went to postprocessing and then, they were exported in standard tessellation language (STL) file format for their subsequent analysis. Moreover, a digital chronometer was used to record the scanning timing (Casio HS-80TW-1EF, Casio Computer Co. Ltd., Tokyo, Japan).

**Figure 4:**
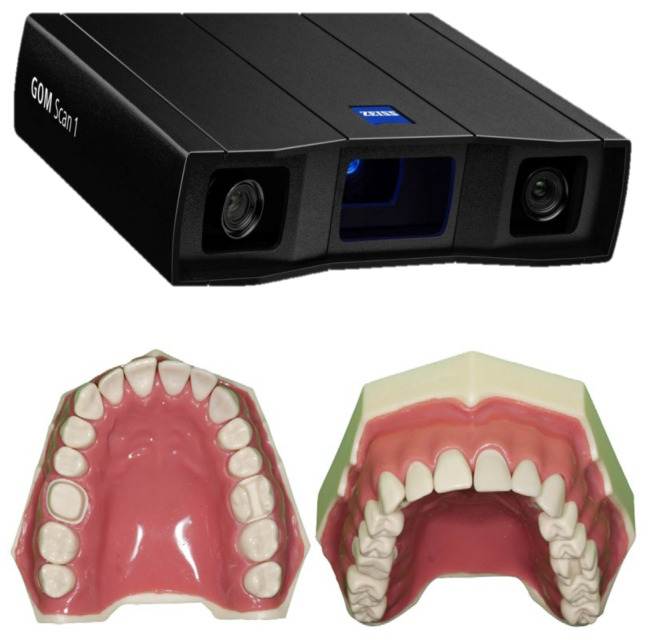
The master model was scanned with an industrial high-resolution reference scanner to obtain a highly accurate digitized reference model GOM Scan 1 (GOM, Zeiss Group, Braunschweig, Germany).

The master model was scanned with an industrial high-resolution reference scanner to obtain a highly accurate digitized reference model GOM Scan 1 (GOM, Zeiss Group, Braunschweig, Germany), as shown in Figure 4.

All the obtained digital models were compared with the reference model. The software GOM Inspect Professional (GOM, Zeiss Group, Braunschweig, Germany) was used to compare the STL files, using a best fit alignment. GOM Inspect Professional is a 3D point cloud (and triangular mesh) processing software. It was originally designed to perform comparison between two dense 3D points clouds (such as the ones acquired with a laser scanner) or between a point cloud and a triangular mesh. It relies on a specific octree structure dedicated to this task. GOM Inspect allowed us to automatically compare the tested scans with the scan of the reference model. In other words, first of all, the master model was cut considering only the teeth. Then, all the scans were uploaded. Consequentially, the software automatically aligned, compared and analyzed all the tested scans, taking into account only the selected areas of the master model.

The discrepancy between the master model and each scan, both when overestimating and when underestimating the areas, was recorded for each of the 6 study groups. The total mean discrepancy calculated from the average of the mean internal and external discrepancies corresponded to trueness, considering the master model and each scan. The total mean discrepancy calculated from the average of the mean internal and external discrepancies corresponded to precision, considering a random scan of a scan group and each scan of that scan group (excluding the considered one).

The results were evaluated using R statistical software. Descriptive statistic values were given as mean ± standard deviation (SD) (all values in μm). The level of Type I error was calibrated at a = 0.05. To test absolute differences in trueness, precision, and time, the Kruskal–Wallis rank sum test was used to evaluate the difference among the scanners. The Wilcoxon signed rank test was used on the signed differences (between each scan and the reference file) to evaluate whether the scanners overestimated or underestimated the size of the reference file by testing whether the center of each scanner’s distribution will be statistically different from zero.

## 3. Results

Overall, a total of *n* = 300 scans were performed. The results of the study in terms of both trueness and precision are shown in Table 1.

### 3.1. Scanning Techniques

#### 3.1.1. Dexis IS 3700

Following the first scanning technique, the trained operator obtained a trueness value of 43.8 ± 5.8 μm and a precision value of 71.0 ± 44.1 μm. The average time taken to scan was 184.6 ± 23.0 s. The unexperienced operator recorded a trueness value of 38.8 ± 8.6 μm and a precision value of 74.0 ± 45.2 μm. The average time taken to scan was 248.6 ± 5.7 s. Considering both the trueness and the precision, a statistically significative difference was not observed between the two operators (*p* > 0.05). On the contrary, a statistically significative difference was recorded when considering the scanning timing (*p* < 0.01). The cutting-out scanning technique did not show any statistically significative differences in terms of both trueness and precision, when compared to the first scanning technique (*p* > 0.05).

Considering the second and the third scanning techniques, a trueness value of 32.4 ± 4.2 μm and 47.8 ± 10.7 μm was, respectively, recorded, while a precision value of 57.0 ± 31.9 μm and 59.0 ± 33.8 μm was, respectively, observed. The mean scanning time was 181.2 ± 16.9 s following the second scanning technique, while it was 170.5 ± 21.2 s following the third one. The statistical analysis showed statistically significant differences in trueness when comparing the first scanning technique to the second one (*p* < 0.01) and the second scanning technique to the third one (*p* < 0.05).

#### 3.1.2. iTero 5D

Considering the first scanning technique, the trained operator obtained a trueness value of 36.6 ± 13.6 μm and a precision value of 97.9 ± 38.4 μm. The average time taken to scan was 229.4 ± 26.4 s. The unexperienced operator recorded a trueness value of 39.2 ± 18.3 μm and a precision value of 60.9 ± 23.7 μm. The average time taken to scan was 317.6 ± 76.5 s. Considering both the trueness and the precision, a statistically significative difference was not observed between the two operators (*p* > 0.05). On the contrary, a statistically significative difference was recorded when considering the scanning timing (*p* < 0.01). The cutting-out scanning technique did not show any statistically significative difference in terms of trueness (*p* > 0.05), but it did in terms of precision (*p* < 0.01), when compared to the first scanning technique. The cutting-out scanning technique was more precise than the scanning technique 1 (55.3 ± 22.1 μm and 97.9 ± 38.4 μm, respectively).

Taking into account the second and the third scanning techniques, a trueness value of 30.1 ± 3.4 μm and 43.6 ± 8.6 μm was, respectively, recorded, while a precision value of 66.2 ± 22.8 μm and 78.3 ± 33.3 μm was, respectively, observed. The mean scanning time was 204.5 ± 15.1 s following the second scanning technique, while it was 211.9 ± 24.4 s following the third one. The statistical analysis showed statistically significant differences in trueness when comparing the second scanning technique to the third one (*p* < 0.001) and in precision when considering the first scanning technique and the second one (*p* < 0.05) and the first and the third one (*p* < 0.05).

#### 3.1.3. Medit i700

When considering the first scanning technique, the trained operator recorded a trueness value of 24.4 ± 2.1 μm and a precision value of 21.4 ± 12.9 μm. The average time taken to scan was 143.6 ± 5.6 s. The unexperienced operator recorded a trueness value of 35.6 ± 3.7 μm and a precision value of 36.8 ± 23.2 μm. The average time taken to scan was 228.2 ± 33.6 s. Considering the trueness, a statistically significative difference was observed between the two operators (*p* < 0.001). A statistically significative difference was also recorded when considering the scanning timing (*p* < 0.01). The cutting-out scanning technique did not show any statistically significative differences in terms of both trueness and precision, when compared to the first scanning technique (*p* > 0.05).

Considering the second and the third scanning techniques, a trueness value of 23.0 ± 1.6 μm and 24.0 ± 2.7 μm was, respectively, observed, while a precision value of 30.0 ± 18.0 μm and 26.6 ± 18.6 μm was, respectively, recorded. The mean scanning time was 147.7 ± 32.3 s following the second scanning technique, while it was 127.6 ± 7.2 s following the third one. The statistical analysis showed statistically significant differences in trueness when comparing the first scanning technique to the third one (*p* < 0.01) and the second scanning technique to the third one (*p* < 0.05).

#### 3.1.4. Omnicam v 5.1.3

Following the first scanning technique, the trained operator obtained a trueness value of 43.0 ± 5.8 μm and a precision value of 26.9 ± 15.8 μm. The average time taken to scan was 191.0 ± 20.7 s. The unexperienced operator recorded a trueness value of 51.7 ± 5.9 μm and a precision value of 25.2 ± 10.1 μm. The average time taken to scan was 256.9 ± 22.4 s. Considering both the trueness and the precision, a statistically significative difference was not observed between the two operators (*p* > 0.05). On the contrary, a statistically significative difference was recorded when considering the scanning timing (*p* < 0.01). The cutting-out scanning technique did not show any statistically significative differences in terms of both trueness and precision, when compared to the first scanning technique (*p* > 0.05).

Considering the second and the third scanning techniques, a trueness value of 49.1 ± 7.3 μm and 42.0 ± 4.9 μm was, respectively, recorded, while a precision value of 40.3 ± 16.9 μm and 31.5 ± 14.1 μm was, respectively, observed. The mean scanning time was 125.2 ± 11.9 s following the second scanning technique, while it was 154.1 ± 33.5 s following the third one. The statistical analysis showed statistically significant differences in trueness when comparing the first scanning technique to the third one (*p* < 0.01) and the second scanning technique to the third one (*p* < 0.05).

#### 3.1.5. Primescan v 5.1.3

Considering the first scanning technique, the trained operator obtained a trueness value of 28.9 ± 7.0 μm and a precision value of 43.1 ± 19.8 μm. The average time taken to scan was 117.3 ± 19.0 s. The unexperienced operator recorded a trueness value of 33.5 ± 5.8 μm and a precision value of 31.3 ± 14.7 μm. The average time taken to scan was 201.0 ± 7.1 s. Considering both the trueness and the precision, a statistically significative difference was not observed between the two operators (*p* > 0.05). On the contrary, a statistically significative difference was recorded when considering the scanning timing (*p* < 0.01). The cutting-out scanning technique did not show any statistically significative differences in terms of both trueness and precision, when compared to the first scanning technique (*p* > 0.05).

Following the second and the third scanning techniques, a trueness value of 25.9 ± 4.3 μm and 25.7 ± 4.4 μm was, respectively, recorded, while a precision value of 38.0 ± 16.1 μm and 26.8 ± 13.7 μm was, respectively, observed. The mean scanning time was 163.9 ± 27.2 s following the second scanning technique, while it was 142.6 ± 37.0 s following the third one. The statistical analysis showed statistically significant differences in trueness when comparing the second scanning technique to the third one (*p* < 0.05).

#### 3.1.6. Trios 4

Following the first scanning technique, the trained operator obtained a trueness value of 53.8 ± 14.9 μm and a precision value of 97.3 ± 41.8 μm. The average time taken to scan was 142.1 ± 15.9 s. The unexperienced operator recorded a trueness value of 58.6 ± 20.4 μm and a precision value of 101.8 ± 53.6 μm. The average time taken to scan was 163.8 ± 30.0 s. No statistically significative difference was observed between the two operators (*p* > 0.05). The cutting-out scanning technique did not show any statistically significative differences in terms of both trueness and precision, when compared to the first scanning technique (*p* > 0.05).

Taking into account the second and the third scanning techniques, a trueness value of 42.2 ± 17.0 μm and 39.7 ± 6.6 μm was, respectively, recorded, while a precision value of 83.5 ± 46.8 μm and 61.4 ± 28.9 μm was, respectively, observed. The mean scanning time was 148.1 ± 12.8 s following the second scanning technique, while it was 144.5 ± 14.0 s following the third one. The statistical analysis showed statistically significant differences in both trueness and precision when comparing the first scanning technique to the third one (*p* < 0.05).

### 3.2. IOSs Inter-Group Comparison according to the Tested Scanning Strategies

**Figure 5:**
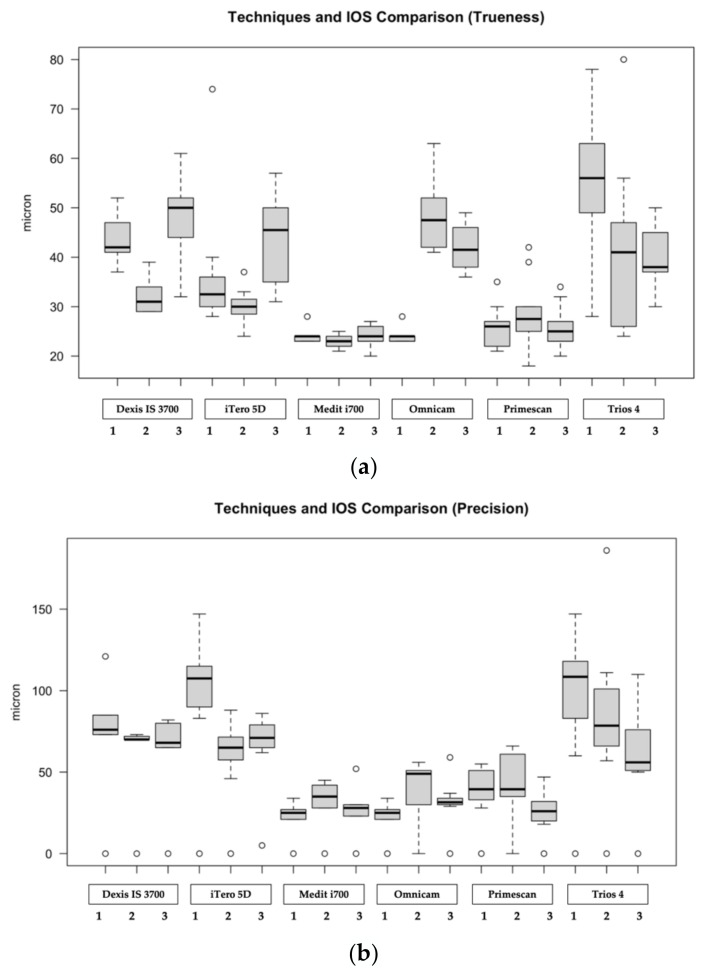

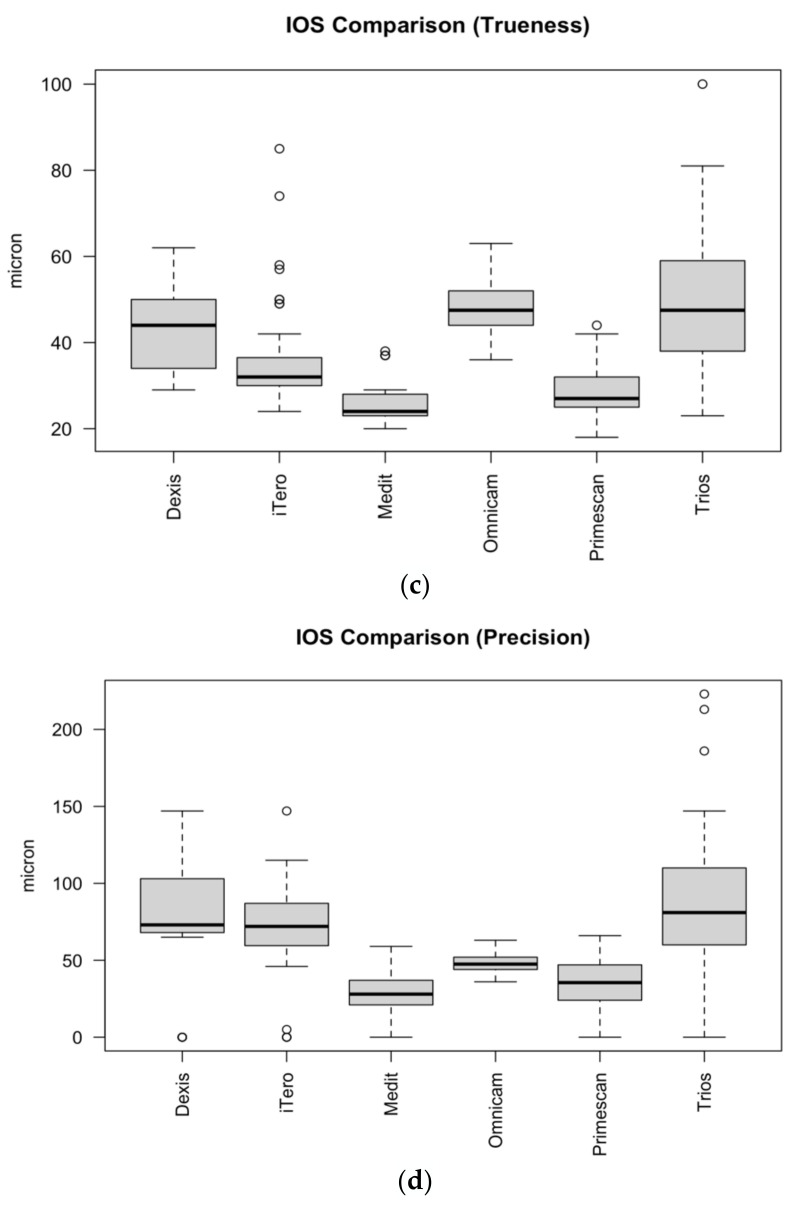
Graphical representation of the results of IOSs according to the studied scanning strategies: (**a**) Boxplot referring to trueness; (**b**) Boxplot referring to precision; (**c**) trueness related pooled data according to the studied IOSs; (**d**) precision related pooled data according to the studied IOSs. Data are based on a five number summary (“minimum” value, first quartile [Q1], median, third quartile [Q3] and “maximum” value).

A comparison was also performed among IOSs according to the used scanning techniques. Overall, according to the first scanning technique, the Medit i700 scanner obtained the best values both in terms of trueness (24.4 ± 2.1 μm) and precision (21.4 ± 12.9 μm) compared to other IOSs (*p* < 0.05), Primescan excluded (*p* > 0.05). In terms of scanning time, Primescan was the fastest scanner (117.3 ± 19.0 s), *p* < 0.05. As well, when considering the second scanning technique, Medit i700 obtained the best results in terms of trueness (23.0 ± 1.6 μm) and precision (30.0 ± 18.0 μm), *p* < 0.05. Omnicam was the fastest IOS (125.2 ± 11.9 s), *p* < 0.05. Additionally, for the third scanning technique, Medit i700 obtained the best values in terms of trueness (24.0 ± 2.7 μm), while Primescan recorded the best values in terms of precision (26.8 ± 13.7 μm), *p* < 0.05. Medit i700 was the fastest scanner (127.6 ± 7.2 s), *p* < 0.05. Once the data were pooled, Medit i700 and Primescan obtained the best results in terms of both trueness and precision, showing no statistically significative differences (*p* > 0.05). Figure 5 graphically outlines the results of IOSs according to the studied scanning strategies and IOSs.

Interestingly, the scanning strategy suggested by the manufacturers did obtain the best results in terms of trueness in none of the tested IOSs. On the contrary, the latter recorded the best results in terms of precision with iTero5D, Medit i700, and Omnicam. Table 2 shows the results obtained according to IOS and the scanning strategy, within the same study group (e.g., Dexis, iTero, Medit, Omnicam, Primescan, Trios).

## 4. Discussion

A total of *n* = 300 scans were performed, aiming at investigating the accuracy of six different IOSs, comparing different scanning strategies and verifying whether the scanning strategies were operative-sensitive. Overall, despite the fact that statistically significant differences were observed in the analyses, none of them recorded a full dental arch distortion higher than 110 μm. This result was in accordance with those of a recent study where the same IOSs showed a distortion of less than 100 μm [20].

The followed scanning strategy was an important parameter to consider when a scan of the dental arch is taken, as it could play an important role in the success of the scan itself, in terms of accuracy and time. Scanning strategies are usually specific to the different IOSs as they are based on different technologies [16]. Each IOS manufacturer recommends a different scanning technique; however, there is no evidence on which is the best of the techniques suggested by the different companies [13]. Various studies investigated the impact of scanning pattern on the accuracy of the scan, with some discordant results. In accordance with the results of this report, other studies also showed that, despite the high accuracy of each IOS, some scanning strategies performed better than others in terms of trueness and precision [21]. Surprisingly, in the present study, the scanning strategy suggested by the manufacturer did not obtain the best results in terms of trueness in none of the tested IOSs. On the contrary, scanning technique 1 obtained the best results in terms of precision with iTero 5D, Medit i700 and Omnicam. A recent study investigated the accuracy of full-arch intraoral scans obtained by different scan strategies with the segmental scan and merge methods: if dental arches are scanned segmentally in two parts, the accuracy is comparable with the one-time scan method. Conversely, when scanning is performed in more than two segments, the accuracy of full-arch image decreases, especially in the intermolar distance evaluation [22].

The rationale for the assessment of the accuracy of the cutting-out technique was that during the daily clinical practice, it may be necessary to cut out some existing cast areas and perform another scan of the same area, due to several reason (e.g., practical, modification of dental preparation, scan correction). Therefore, it was deemed necessary to verify if the cutting-out procedure affects the accuracy of the scan. In accordance with a recent study, the cutting-out technique did not obtain clinical significative differences in terms of both trueness and precision, when compared to scanning technique 1 [23]. It could be speculated that, if necessary, re-scanning a specific area of the dental arch does not affect the overall accuracy of the scan itself. However, in contrast with the results of the present study, other studies showed how cutting off and rescanning procedures affected the accuracy of intraoral scanning, as a consequence of the stitching process [24,25].

It is crucial to consider that to use IOS in clinical practice, it is necessary to acquire specific skills and familiarity with this kind of technology [26,27,28]. In addition, manufacturers provide different and specific scanning strategies for each different device and clinical situation, further complicating the correct use of IOS. The experience level of the operator was demonstrated to play an important role in IOS working-time, as showed elsewhere [29]. Additionally, in the present study, the operator with no experience with IOS recorded significantly longer times than the experienced operator in performing the complete arch scans. A recent study investigated the impact of operators’ experience in terms of both scanning time and accuracy [30]. In accordance with the results of this study, it was found that the most experienced operator spent less time than the unexperienced one in scanning the complete dental arch, while operator’s experience had only a small influence on the scan accuracy. In fact, the difference between the two operators was observed only for the scanning timing, as there were no statistically significative differences in terms of accuracy. This datum was in contrast with the results of another study that showed that operator experience and scanner type play an important role in accuracy and scanning time [31]. However, as reported elsewhere, with an adequate training, the needed time for the intraoral scan can be easily shortened [32]. The degree of experience, therefore, is directly correlated with the times of scanning while it did not seem to improve the accuracy of the scans. Young clinicians, with a greater affinity for the digital world, will find it easier to use intraoral scanners and related software in their clinical practice compared to dentists with less experience and passion for technological innovations [33,34]. In addition, the use of IOS is preferred and perceived as easier than the conventional technique among undergraduate dental students with no impression-making experience [35].

The use of an in vitro model may have limited the results of the study as it did not correspond perfectly to the results obtainable in vivo. However, the choice to conduct an in vitro study was supported by the need to assess accuracy, which cannot be easily measured in vivo, due to the necessity to have a virtual reference model on which to superimpose, through reverse engineering software, the obtained intraoral scans. To date, a virtual reference model can only be created by indirect extraoral methods, using sophisticated industrial devices. Therefore, the calculation of the accuracy in vivo may be tricky due to the anatomical structures that may obstacle the action of the industrial scanner. Another limitation of the present report was that this study did not examine all the intraoral scanners on the market. Therefore, the results of this study were not exhaustive. However, the main IOS systems used in dental practice were tested. In addition to this, it was necessary to respect the availability of different companies in providing the various devices for carrying out the study. Not all the scanning techniques proposed in the literature were tested. Three were selected, each having potential advantages over the others. The first scanning technique was chosen because it represented the strategy recommended by the manufacturers for each specific device and it was assumed to be the most tested and suitable for the correct use of IOS; the second scanning technique was chosen because, being less complex and articulated than the others, it was deemed to be time-saving and clinically advantageous; the third scanning technique was tested as it was demonstrated to be the most accurate one, as showed elsewhere [10]. Finally, considering the study protocol, the unexperienced operator performed the scans using only the technique recommended by the manufacturer (Technique 1). The rationale of it was that when someone approaches something new, the novice should start the learning curve following a widely tested and validated protocol.

## 5. Conclusions

The examined IOSs showed statistically significant differences in terms of trueness and precision. However, clinically, these differences were deemed to be not relevant since the recorded accuracy values were below the threshold of clinical acceptability. Overall, Medit i700 and Primescan recorded the best results in terms of trueness and precision (*p* < 0.05). The tested scanning strategies were proven to be a factor influencing the accuracy of intraoral scanners, even though, clinically, no technique was found to be the best one. On the contrary, scanning strategies are time-sensitive (*p* < 0.05). Considering the experience of the operators, scanning strategies are not operative sensitive in terms of accuracy. However, they are in terms of time spent in scanning.

## Figures and Tables

**Table 1:** This table shows the results of the present study according to: scanning technique, the operator and IOS. Data are expressed in micron and seconds.

Intraoral Scanner	Trueness (μm)	Precision (μm)	Time (s)
Scanning Technique 1 (Expert)			
Dexis IS 3700	43.8 ± 5.8	71.0 ± 44.1	184.6 ± 23.0
iTero 5D	36.6 ± 13.6	97.9 ± 38.4	229.4 ± 26.4
Medit i700	24.4 ± 2.1 *	21.4 ± 12.9 *	143.6 ± 5.6
Omnicam	43.0 ± 5.8	26.9 ± 15.8 *	191.0 ± 20.7
Primescan	28.9 ± 7.0 *	43.1 ± 19.8	117.3 ± 19.0 *
Trios 4	53.8 ± 14.9	97.3 ± 41.8	142.1 ± 15.9
Scanning Technique 1 (Not Expert)			
Dexis IS 3700	38.8 ± 8.6	74.0 ± 45.2	248.6 ± 5.7
iTero 5D	39.2 ± 18.3	60.9 ± 23.7	317.6 ± 76.5
Medit i700	35.6 ± 3.7 *	36.8 ± 23.2	228.2 ± 33.6
Omnicam	51.7 ± 5.9	25.2 ± 10.1 *	256.9 ± 22.4
Primescan	33.5 ± 5.8 *	31.3 ± 14.7 *	201.0 ± 7.1
Trios 4	58.6 ± 20.4	101.8 ± 53.6	163.8 ± 30.0 *
Cut-Out Re-scan Technique			
Dexis IS 3700	51.4 ± 6.6	109.2 ± 61.5	267.7 ± 21.4
iTero 5D	31.9 ± 3.8	55.3 ± 22.1	332.6 ± 25.1
Medit i700	23.2 ± 3.0 *	22.4 ± 13.9 *	208.2 ± 7.7
Omnicam	49.40 ± 2.9	33.5 ± 17.7	276.9 ± 21.3
Primescan	28.3 ± 4.2 *	33.9 ± 18.4	170.1 ± 19.4 *
Trios 4	48.1 ± 13.9	85.3 ± 55.9	206.1 ± 13.8
Scanning Technique 2			
Dexis IS 3700	32.4 ± 4.2	57.0 ± 31.9	181.2 ± 16.9
iTero 5D	30.1 ± 3.4	66.2 ± 22.8	204.5 ± 15.1
Medit i700	23.0 ± 1.6 *	30.0 ± 18.0 *	147.7 ± 32.3
Omnicam	49.1 ± 7.3	40.3 ± 16.9	125.2 ± 11.9 *
Primescan	25.9 ± 4.3 *	38.0 ± 16.1	163.9 ± 27.2
Trios 4	42.2 ± 17.0	83.5 ± 46.8	148.1 ± 12.8
Scanning Technique 3			
Dexis IS 3700	47.8 ± 10.7	59.0 ± 33.8	170.5 ± 21.2
iTero 5D	43.6 ± 8.6	78.3 ± 33.3	211.9 ± 24.4
Medit i700	24.0 ± 2.7 *	26.6 ± 18.6 *	127.6 ± 7.2 *
Omnicam	42.0 ± 4.9	31.5 ± 14.1	154.1 ± 33.5
Primescan	25.7 ± 4.4 *	26.8 ± 13.7 *	142.6 ± 37.0
Trios 4	39.7 ± 6.6	61.4 ± 28.9	144.5 ± 14.0

* *p* < 0.05.

**Table 2:** Table 2 shows the results obtained according to IOS and the scanning strategy, within the same study group.

	Trueness (Mean ± Sd, μm)	Scanning Technique	*p*-Value	Precision (Mean ± Sd, μm)	Scanning Technique	*p*-Value	Time (s)	Scanning Technique	*p*-Value
Dexis	32.4 ± 4.2	2	<0.05	57.0 ± 31.9	2	n.s.	170.5 ± 21.2	3	n.s.
iTero	30.1 ± 3.4	2	n.s.	60.9 ± 23.7	1	<0.05	204.5 ± 15.1	2	<0.05
Medit	23.0 ± 1.6	2	n.s.	21.4 ± 12.9	1	n.s.	127.6 ± 7.2	3	<0.05
Omnicam	42.0 ± 4.9	3	<0.05	25.2 ± 10.1	1	n.s.	125.2 ± 11.9	2	<0.01
Primescan	25.7 ± 4.4	3	n.s.	26.8 ± 13.7	3	n.s.	117.3 ± 19.0	1	<0.05
Trios	39.7 ± 6.6	3	<0.05	61.4 ± 28.9	3	n.s.	142.1 ± 15.9	1	n.s.

Abbreviations: n.s. = not significative.

## Data Availability

The data sets generated and/or analyzed during the present study are available from the corresponding author on reasonable request.
